# α-Lipoic Acid Protects against Cyclosporine A-Induced Hepatic Toxicity in Rats: Effect on Oxidative Stress, Inflammation, and Apoptosis

**DOI:** 10.3390/toxics10080442

**Published:** 2022-08-02

**Authors:** Eman M. El-Mancy, Dalia Mahmoud Abdelmonem Elsherbini, Rasha Hamed Al-Serwi, Mohamed El-Sherbiny, Gehan Ahmed Shaker, Abdel-Moneim Hafez Abdel-Moneim, Eman T. Enan, Nehal M. Elsherbiny

**Affiliations:** 1 Deanship of Common First Year, Jouf University, P.O. Box 2014, Sakaka 42421, Saudi Arabia; eelmancy@ju.edu.sa; 2 Zoology Department, Faculty of Women for Arts, Science and Education, Ain Shams University, Cairo 11511, Egypt; 3 Department of Clinical Laboratory Sciences, College of Applied Medical Sciences, Jouf University, P.O. Box 2014, Sakaka 42421, Saudi Arabia; dmelsherbini@ju.edu.sa; 4 Department of Anatomy, Faculty of Medicine, Mansoura University, Mansoura 35516, Egypt; 5 Department of Basic Dental Sciences, College of Dentistry, Princess Nourah bint Abdulrahman University, P.O. Box 84428, Riyadh 11671, Saudi Arabia; rhalserwi@pnu.edu.sa; 6 Department of Basic Medical Sciences, College of Medicine, AlMaarefa University, P.O. Box 71666, Riyadh 11597, Saudi Arabia; 7 Department of Medical Physiology, Faculty of Medicine, Mansoura University, Mansoura 35516, Egypt; gehanshaker@mans.edu.eg (G.A.S.); a.elmonem@qu.edu.sa (A.-M.H.A.-M.); 8 Department of Medical Physiology, Faculty of Medicine, Qassim University, Buraydah 51452, Saudi Arabia; 9 Department of Pathology, Faculty of Medicine, Mansoura University, Mansoura 35516, Egypt; emanenan@mans.adu.eg; 10 Department of Pharmaceutical Chemistry, Faculty of Pharmacy, University of Tabuk, Tabuk 71491, Saudi Arabia; 11 Department of Biochemistry, Faculty of Pharmacy, Mansoura University, Mansoura 35516, Egypt

**Keywords:** cyclosporine A, liver, oxidative stress, inflammation, apoptosis

## Abstract

The clinical application of cyclosporine A (CsA) as an immunosuppressive agent is limited by its organ toxicity. We aimed to evaluate the effectiveness of α-lipoic acid against CsA-induced hepatotoxicity and to delineate the underlying molecular mechanisms. Male Wistar rats (*n* = 24, 8 per each group) received the vehicle, CsA (25 mg/kg) and/or ALA (100 mg/kg, p.o.) for 3 weeks. Biochemical markers of liver function (serum ALT, AST, ALP < GGT), oxidative stress (MDA, TAC, SOD, GSH, Nrf2/HO-1), inflammation (NF-κB, CD68, iNOS, NO, COX-2), and apoptosis (caspase-3) were assessed in serum and tissue. Liver histological analysis using H&E and Sirius red was performed. The development of liver injury in CsA-treated animals was indicated by elevated levels of liver enzymes, oxidants/antioxidants imbalance, inflammatory cells infiltration, up-regulated expression of inflammatory mediators, and apoptosis. These changes were associated with altered architecture of hepatic cells and fibrous connective tissue. ALA co-administration protected against CsA-induced liver damage and ameliorated biochemical changes and cellular injury. In conclusion, ALA demonstrated hepatoprotective potential against CsA-induced liver injury through combating oxidative stress, inflammation, and apoptosis, highlighting ALA as a valuable adjunct to CsA therapy.

## 1. Introduction

The increased rate of organ transplantation and incidence of autoimmune diseases worldwide have resulted in wide application of immunosuppressive agents for better clinical outcomes [1]. Cyclosporine A (CsA) is an immunosuppressant peptide widely used in autoimmune diseases and transplant operations. It acts by a unique mechanism to inhibit the T cell’s lymphokine production and signal transduction. It forms an active complex with cyclophilin that inhibits calcineurin activity, thereby suppressing the activation of the nuclear factor of activated T cell (NFAT). Activation and nuclear translocation of NFAT are needed for the transcription of many cytokines which are essential for T cell proliferation and maturation, such as interleukin-2 (IL-2). Additionally, CsA increases T cells cytosolic calcium, which increases vulnerability of the cells to oxidative stress-induced cell death [2]. However, its clinical use is restricted due to side effects that affect various body organs including the kidney, spleen, liver, heart, and neurons. In this context, liver dysfunction was reported in CSA-treated rats. It is characterized by hyper-bilirubinemia, hypoproteinemia, cholestasis, increased transaminases, and alkaline phosphatase activities. Various studies reported the role of oxidative stress and inflammation in CsA-induced organ toxicity. This was further ascertained by the ameliorative effects reported by antioxidant and anti-inflammatory agents in experimental models of CsA-induced organ toxicity [3,4].

Alpha-lipoic acid (ALA, 5-(1,2-dithiolan-3-yl) pentanoic acid) is a powerful antioxidant compound with a reportedly good safety profile. ALA is a natural compound that can be found in a variety of dietary sources or endogenously synthetized. It is also known as thioctic acid and is widely distributed in the cellular membranes of both prokaryotes and eukaryotes. Endogenously, it is involved in the metabolism of carbohydrates and fats mainly via acting as a cofactor for many mitochondrial enzyme complexes [5]. Additionally, it is reported to suppress oxidative stress and inflammation in various diseases due to its metal chelating activity and ability to restore a reduced form of intracellular antioxidants, including glutathione (GSH), vitamin E, and vitamin C, against environmental pollutants, such as heavy metals [6]. Indeed, ALA was proposed to offer more protection from oxidative damage compared to the endogenous reduced/oxidized glutathione system. This could be attributed to its high redox potential (−320 mV). Additionally, its specific amphiphilic characteristics could allow for better distribution between intra- and extra-cellular compartments [7]. Due to the presence of a chiral center at the C6 location, ALA exists as two enantiomers (R and S), however only the R isomer can be endogenously found [8]. ALA is commercially available as dietary and antiaging supplements and in multivitamin formula. Specifically, in liver, ALA was reported to suppress the hepatic stellate cell activation and protect against hepatotoxicity triggered by various toxins [9]. Indeed, ALA protects against high-fat diet-induced fatty liver [10], concanavalin A-induced hepatitis [11], acetaminophen-induced liver damage [12], lipopolysaccharide induced-acute liver injury [13], and carbon–tetrachloride-induced liver cirrhosis [14]. Moreover, ALA supplementation has been previously reported to enhance erythrocytic antioxidant defense mechanism and attenuate renal injury in CsA-treated rats [15]. Therefore, the present study aims to evaluate the hepatoprotective effect of ALA in CsA-treated rats and to evaluate the underlying mechanism.

## 2. Materials and Methods

### 2.1. Chemicals

CsA was presented in the form of ampoules under the traditional name Sandimmun and was provided by Novartis Pharma (Basel, Switzerland). It was presented as a clear, yellow liquid supplied in 1 mL ampoules containing 50 mg/mL and was further diluted with olive oil to a final concentration of 50 mg/4 mL (25 mg/2 mL).

Alpha-Lipoic acid (ALA) (Sigma Chemical Company, St. Louis, MO, USA) was purchased in the form of a yellow powder and was suspended in physiological saline solution (0.9% NaCl) to a final concentration of 100 mg/2 mL.

### 2.2. Animals

A total of thirty-two adult male albino rats of Wistar strain (weighing 180 ± 20 g, 12–14 weeks age) were obtained from an animal house in the Medical Research Center (MRC), Faculty of Medicine, Ain Shams University. The animals were acclimatized to the laboratory conditions for a period of 2 weeks. They were housed and maintained at an ambient temperature of 25 ± 2 °C, 50 ± 20% relative humidity, 12-h light/12-h dark cycle, and were given a standard rat feed and water ad libitum. The design of the experiments was conducted in accordance with the guidelines provided by the Experimental Animal Laboratory and approved by the Animal Care and Use Committee of Mansoura University, Mansoura, Egypt (approval number R.22.07.1760).

### 2.3. Experimental Design

Rats were randomly divided into four equal groups of eight rats each:

Group I (Control) were pre-treated orally (per os, p.o.) with saline (2 mL/kg b.w.) 1 h prior to oral treatment with olive oil (2 mL/kg b.w.), once a day for 21 days.

Group II (ALA-treated group) received ALA (100 mg/kg b.w., p.o.), once a day for 21 days

Group III (CsA-treated group) received CsA (25 mg/kg b.w., p.o.), once a day for 21 days

Group IV (ALA+ CsA-treated group) were pre-treated with ALA (100 mg/kg b. w., p.o.) 1 h before oral treatment by CsA (25 mg/kg b.w.), once a day for 21 days. The experimental procedure and the doses for CsA and ALA were selected based on preliminary experiments and previous studies [1,16,17].

### 2.4. Sampling

At the end of the experimental protocol duration, after 12 h overnight fasting, a blood sample was withdrawn under completely aseptic conditions from the retro-orbital venous plexus using a disposable plastic syringe. The samples were collected into plastic containers and sera were collected after centrifugation at 15,000 rpm for 20 min, then divided into aliquots and stored at −80 °C until used for biochemical investigations: liver function tests (ALT, AST, ALP, and GGT) and oxidative stress markers (MDA, TAC, and NO).

All rats from each group were sacrificed under diethyl ether anesthesia, and the liver was removed for molecular studies. The liquid nitrogen frozen liver tissue samples (30–50 mg in weight) were used for total RNA extraction and real-time qRT-PCR analysis of expression of SOD, Nrf-2, TNF-α, NF-KB and Caspase-3.

### 2.5. Biochemical Investigations

Activities of alanine transaminase enzyme (ALT, Diamond Diagnostic Co., Cairo, Egypt), aspartate transaminase enzyme (AST, Diamond Diagnostic Co., Cairo, Egypt), γ glutamyl transpeptidase (GGT, Qu mica Clínica Aplicada SA, Amposta, Spain), and alkaline phosphatase (ALP, Bio diagnostic, Cairo, Egypt) were estimated according to the manufacturer’s instructions.

Oxidative stress markers were measured in the sera and tissues of all rats. Estimation of the lipid peroxidation product, Malondialdehyde (MDA), was done based on the thiobarbituric acid reaction [18], and the results were expressed as μmol MDA/L. The total antioxidant capacity (TAC) was estimated by the commercially available colorimetric kits (Cat.No. # TA 25 12), which were supplied by Bio-Diagnostics, Dokki, Giza, Egypt and used according to the method previously reported by Koracevic et al. [19]. The results were expressed as mmol/L. The serum nitric oxide (NO) was measured by the colorimetric method (Nitric Oxide Assay Kit, Abcam co. ab272517), and the results were expressed as μmol/L. Additionally, activity of superoxide dismutase (SOD) and catalase (CAT) enzymes and the level of reduced glutathione (GSH) were evaluated in hepatic tissues by colorimetric methods using commercially available kits (Bio-Diagnostics, Dokki, Giza, Egypt).

### 2.6. Real Time Polymerase Chain Reaction

Total RNA from rat liver tissue samples (~25 mg) was extracted using Trizol Reagent (Invitrogen) according to the manufacturer’s instructions and stored at −80 °C. The concentration and purity of the total isolated RNA were determined by Nanodrop spectrophotometry. Reverse transcription reaction for cDNA synthesis was performed with ~200 ng total RNA using the Maxima First Strand cDNA Synthesis Kit (Thermo Scientific, USA, cat No. #K1641). The rat liver mRNA expressions of SOD, caspase-3, Nrf-2, TNF-α, and NF-κB, were quantified by real-time PCR using the Applied Biosystem 7500, real-time PCR detection system (Life Technology, Carlsbad, CA, USA) with “HERA^PLUS^ SYBR^®^ Green qPCR Master Mix” (2X) (Willowfort, UK, cat. No. WF10308001). Reaction mixtures were incubated for 10 min at 95 °C, followed by 40 cycles of 15 s at 95 °C, and 30 s at 60 °C. The primer sequences used were rat SOD1: forward, 5′-TTTTGCTCTCCCAGGTTCCG-3′; reverse, 5′-CCCATGCTCGCCTTCAGTTA-3 [20], rat caspase-3: forward, 5′-GTGGAACTGACGATGATATGGC-3′; reverse, 5′-CGCAAAGTGACTGGATGAACC-3′ [21], rat Nrf-2: forward, 5′-GGGCAAAAGCTCTCCATATTCC-3′; reverse 5′-GAGCGGCAACTTTATTCTTCCC-3′) [22], rat TNF-α: forward, 5′-CTGGCGTGTTCATCCGTTC-3′; reverse, 5′-GGCTCTGAGGAGTAGACGATAA-3′ [23], rat NF-κB: forward, 5′-AGAGCAACCGAAACAGAGAGG-3′; reverse, 5′-TTTGCAGGCCCCACATAGTT-3 [24]. The primers sequences for rat β-actin were 5′-AAGATCCTGACCGAGCGTGG-3′ (Forward); and 5′-CAGCACTGTGTTGGCATAGAGG-3′ (Reverse) [21]. The expression of the analyzed genes was normalized to that of the internal control gene, the β-actin, using the comparative ΔΔCT method.

### 2.7. Histopathological Examination

Two different sections of the liver from each animal in the control and treatment groups were collected (16 sections/group), fixed in 10% neutral buffered formalin, and then the specimens were dehydrated in an ascending series of ethyl alcohol, cleared in xylene, and embedded in paraffin wax. The blocks were prepared to contain single section/block (2 blocks/animal, making total 16 blocks/group). Tissue blocks were sectioned into approximately 5 μm thick slices using a rotary microtome. Sections were routinely stained using hematoxylin and eosin (H&E) for histological studies. Evaluation of liver fibrosis was conducted using Sirius red-stained liver sections. Slides were incubated overnight with 0.1% Sirius red (Sigma–Aldrich, UK), treated with 0.01 M hydrochloric acid, and followed by dehydration in serial ethanol concentrations without water. Slides stained with H&E and Sirius red were inspected and captured using ×10 and ×40 objective lens with a camera-aided light microscope. An Olympus® CX41 light microscope was used for examining the sections, which were photographed by its digital camera, Olympus^®^ SC100.

Image J software was used to analyze the area percentage of fibrosis in Sirius red-stained sections [25]. Calibrations were performed with Image J using a straight-line tool at the appropriate calibration (pixel to µm ratio). Measurements were done in the pictures captured by the objective lens of magnification 10X to create images that cover the entire area to be examined. Briefly, to isolate red-stained collagen, we changed the image type to RGB Stack, which yields the gray-scale images of the channels (Image → Type → RGB Stack). In the Green channel, the threshold was set at 0–87 (Image → Adjust → Threshold). We recorded the area, area fraction, limit to threshold, and display label (Analyze → Measure). Two different sections from each animal were analyzed, and from each section, (6–10) different non-overlapping fields were examined (12–20 fields/animal) [26].

### 2.8. Immunohistochemical Studies

For the immunohistochemical study, paraffin sections of 5 µm thickness were prepared and the staining was performed using the labeled Streptavidin–Biotin immunoperoxidase technique according to the manufacturer’s instructions. For IHC quantitative assessment, the Allred score was used. It provides a scale of 0–8 representing the Allred index (0–1 = negative, 2–3 = mild, 4–6 = moderate, and 7–8 = strongly positive). Allred is obtained by the sum of staining intensity grading (0–3) and the positive cell proportion grading (0–5) [27] and is quantified using the QuPath program (0.1.2) [28].

### 2.9. Statistical Analysis

Graphpad prism 8 software was used for statistical analyses and the graphical presentation of data. Quantitative data were initially tested for normality using Shapiro–Wilk’s test, with data being normally distributed if *p* > 0.050. Data are expressed as the mean ± Standard error (SE). Bartlett’s test was used for examining the homogeneity of variances. One-way analysis of variance (ANOVA) was used to compare the quantitative data between the studied groups. Tukey post hoc assessment was used for multiple comparisons when homoscedasticity was met, whereas Games–Howell adjustment was used when homoscedasticity was not met. Significance was considered at *p* values less than 0.05.

## 3. Results

### 3.1. Effect of α-LA on CsA-Induced Changes in Liver Function

As shown in Table 1, rats treated with α-LA demonstrated no changes in liver function indices, indicating the safety of α-LA on normal rats at the selected dose. However, the CsA-treated group showed substantial alterations in liver function indices (ALT, AST, GGT, and ALP) compared to the normal control group. In contrast, administration of α-LA normalized these biochemical indicators of liver function compared to the CsA-treated group.

### 3.2. Effect of α-LA on CsA-Induced Hepatic Cellular Injury and Fibrosis

**Figure 1:**
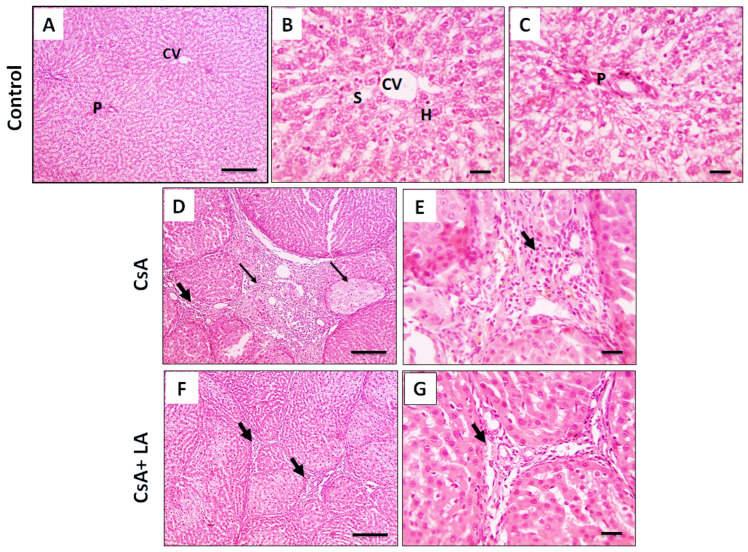
Microscopic pictures of H&E-stained hepatic sections. The control normal group (**A**–**C**) shows regular arrangement of hepatic cords (H) around central veins (CV) with normal portal areas (P) and sinusoids (S). Hepatic sections (**D**,**E**) from the Cyclosporine A (CsA)-treated group show cirrhotic nodules (thin arrows) completely separated by thick fibrous connective tissue (thick arrows) that contains leukocytes, hemosiderin laden macrophages, dilated lymphatics, and proliferated biliary epithelium. Hepatic sections (**F**,**G**) from the Cyclosporine A + lipoic acid (CsA + LA)-treated group show milder lesions characterized by thin fibrous connective tissue deposition (thick arrows) and contains fewer leukocytes, (**A**,**D**,**F**; Scale bar = 100 μm), and (**B**,**C**,**E**,**G**; Scale bar = 50 μm).

H&E staining was used to investigate if α-LA has a protective impact on liver tissue damage induced by CsA. As indicted in Figure 1A–C, hepatic sections from the normal group demonstrated normal arrangement and morphology of the liver cells. The liver cells of the CsA-treated group showed cirrhotic nodules, fibrous connective tissue, inflammatory cells, dilated lymphatics, and proliferated biliary epithelium, Figure 1D,E. However, milder lesions were observed in hepatic sections from αLA + CsA characterized by thin fibrous connective tissue deposition with fewer inflammatory cells, Figure 1F,G.

**Figure 2:**
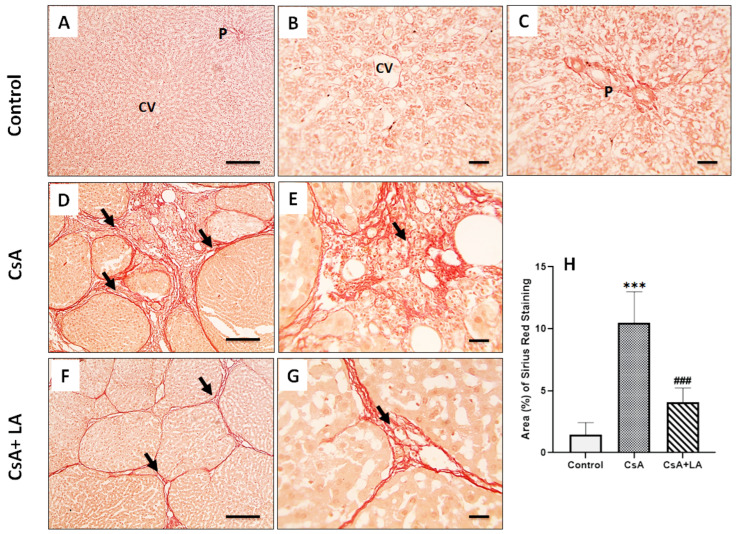
Microscopic pictures of the Sirius red-stained hepatic sections. The control normal group (**A**–**C**) shows no fibrous tissue deposition around central veins (CV) or portal areas (P). Hepatic sections (**D**,**E**) from the CsA-treated group show excessive red-stained fibrous connective tissue deposition (arrows). Hepatic sections (**F**,**G**) from the (CsA + LA) treated group show thin red-stained fibrous connective tissue deposition (arrows), (**A**,**D**,**F**; Scale bar = 100 μm), and (**B**,**C**,**E**,**G**; Scale bar = 50 μm). (**H**) represents a quantitative analysis of liver fibrosis determined by the % collagen deposition calculation from the Sirius red stain. Data are displayed as mean ± SE. *** *p* < 0.001 vs. control group and ^###^ *p* < 0.001 vs. CsA-treated group.

The Sirius red stain was used to observe fibrosis in the different experimental groups. As shown in Figure 2A–C, no fibrous tissue deposition was observed in the normal control group. However, hepatic sections from the CsA-treated group showed excessive connective tissue deposition, Figure 2D,E. On the other hand, administration of α-LA resulted in mild fibrosis, as indicated by thin red-stained fibrous connective tissue deposition in hepatic sections from the CsA + α-LA group, Figure 2F,G.

### 3.3. Effect of α-LA on CsA-Induced Oxidative Stress in Rat Serum and Liver Tissues

**Figure 3:**
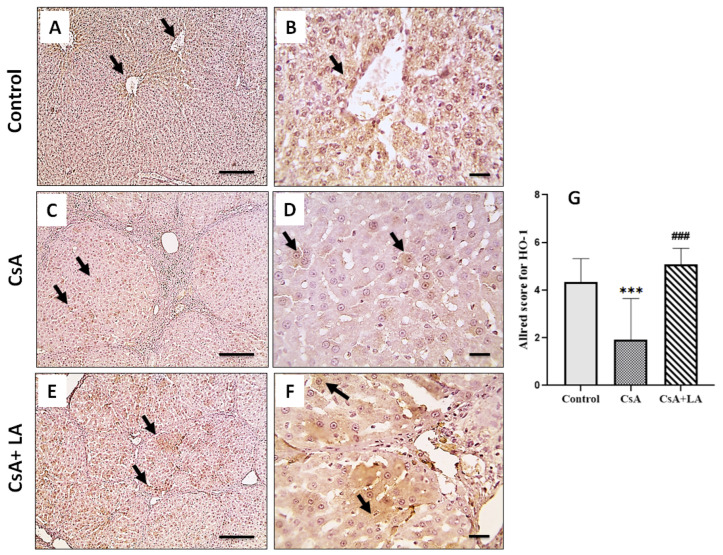
Microscopic pictures of immunostained hepatic sections against HO-1. The control normal group (**A**,**B**) showing many positively stained perisinusoidal liver cells (arrows). Hepatic sections (**C**,**D**) from CsA-treated group show few positively stained parenchymal liver cells (arrows). Hepatic sections (**E**,**F**) from the (CsA + LA)-treated group show significantly increased numbers of positively stained liver cells, perisinusoidal, and in the liver parenchyma (arrows). IHC was counterstained with Mayer’s hematoxylin, (**A**,**C**,**E**; Scale bar = 100 μm), and (**B**,**D**,**F**; Scale bar = 50 μm). (**G**) represents an Allred score for HO-1 cytoplasmic expression. Data are expressed as the mean ± SE (*n* = 8). *** *p* < 0.001 versus the control group, ^###^ *p* < 0.001 versus the CsA group. Allred index (0–1 = negative, 2–3 = mild, 4–6 = moderate, and 7–8 = strongly positive).

Table 2, Table 3 and Table 4 and Figure 3 demonstrate the results of oxidative stress markers in serum and hepatic tissues, respectively from various experimental groups. The CsA-treated group showed a marked increase in the serum and tissue oxidative stress marker MDA, accompanied by a marked reduction in TAC, hepatic tissue mRNA expression of Nrf2 and SOD, SOD and CAT activity, GSH content, and immunostaining of HO-1 when compared to the control group. Treatment with α-LA dampened serum and tissue MDA and restored serum TAC, mRNA expression of Nrf2 and SOD, tissue activity of SOD and CAT, GSH content, and immunostaining of HO-1 in hepatic tissue when compared to the CsA-treated group.

### 3.4. Effect of α-LA on CsA-Induced Inflammation in Rat Liver Tissues

**Figure 4:**
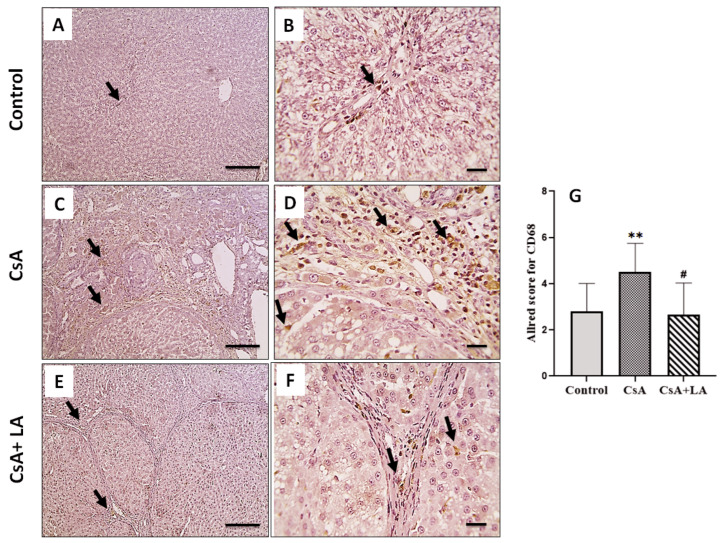
Microscopic pictures of immunostained hepatic sections against CD68. The control normal group (**A**,**B**) shows few positively stained cells in portal areas and sinusoids (arrows). Hepatic sections (**C**,**D**) from the CsA-treated group show markedly increased positively stained cells in portal areas and sinusoids (arrows). Hepatic sections (**E**,**F**) from (CsA + LA) treated group show significantly decreased numbers of positively stained cells in portal areas and sinusoids (arrows). IHC counterstained with Mayer’s hematoxylin, (**A**,**C**,**E**; Scale bar = 100 μm), and (**B**,**D**,**F**; Scale bar = 50 μm). (**G**) represents the Allred score for CD68 cellular expression. Data are expressed as mean ± SE (*n* = 8). ** *p* < 0.01 versus the control group, ^#^ *p* < 0.05 versus the CsA group. Allred index (0–1 = negative, 2–3 = mild, 4–6 = moderate, and 7–8 = strongly positive).

**Figure 5:**
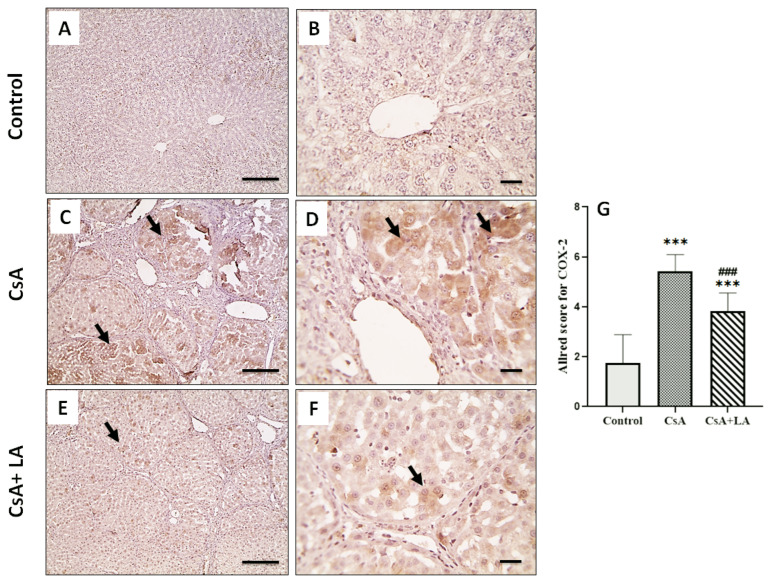
Microscopic pictures of immunostained hepatic sections against COX-2. The control normal group (**A**,**B**) shows negatively stained hepatocytes. Hepatic sections (**C**,**D**) from CsA-treated group show prominent positively stained hepatocytes (arrows). Hepatic sections (**E**,**F**) from (CsA + LA) treated group show significantly decreased numbers of positively stained hepatocytes (arrows). IHC counterstained with Mayer’s hematoxylin, (**A**,**C**,**E**; Scale bar = 100 μm), and (**B**,**D**,**F**; Scale bar = 50 μm). (**G**) represents the Allred score for COX-2 cellular expression. Data are expressed as mean ± SE (*n* = 8). *** *p* < 0.001 versus the control group, ^###^ *p* < 0.001 versus the CsA group. Allred index (0–1 = negative, 2–3 = mild, 4–6 = moderate, and 7–8 = strongly positive).

**Figure 6:**
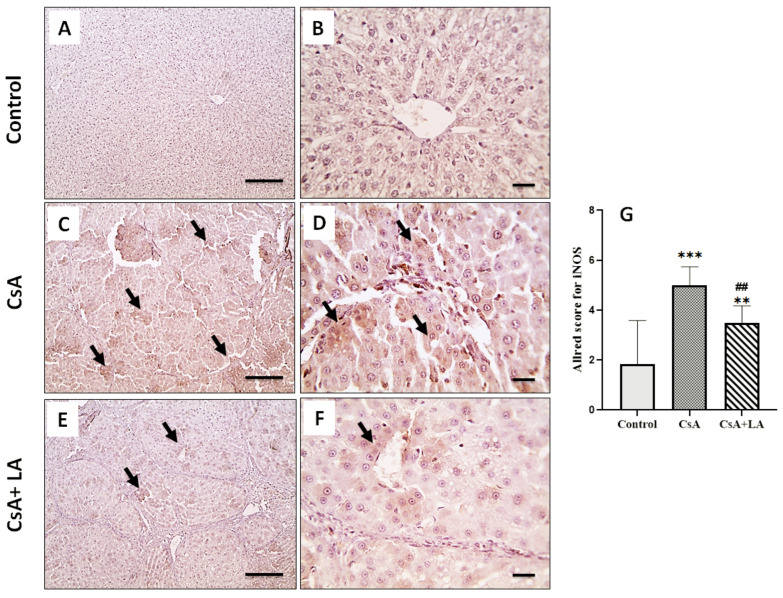
Microscopic pictures of immunostained hepatic sections against iNOS. The control normal group (**A**,**B**) shows negatively stained hepatocytes. Hepatic sections (**C**,**D**) from the CsA-treated group show markedly increased positively stained hepatocytes (arrows). Hepatic sections (**E**,**F**) from the (CsA + LA) treated group show significantly decreased numbers of positively stained hepatocytes (arrows). IHC counterstained with Mayer’s hematoxylin, (**A**,**C**,**E**; Scale bar = 100 μm), and (**B**,**D**,**F**; Scale bar = 50 μm). (**G**) represents the Allred score for iNOS cellular expression. Data are expressed as mean ± SE (*n* = 8). *** *p* < 0.001, ** *p* < 0.01 versus the control group, ^##^ *p* < 0.01 versus the CsA group. Allred index (0–1 = negative, 2–3 = mild, 4–6 = moderate, and 7–8 = strongly positive).

As shown in Table 5 and Figure 4, Figure 5 and Figure 6, CsA treatment is associated with hepatic inflammation, as indicated by a significant increase in the mRNA expression of TNF-α and NF-κB. This was accompanied by a marked increase in CD68, COX-2 and iNOS immunostaining in hepatic tissue and a decrease in serum NO in CsA-treated rats. On the other hand, administration of α-LA along with CsA ameliorated hepatic inflammation, as indicated by a significant downregulation of the mRNA expression of TNF-α and NF-κB, accompanied by marked increase in CD68, COX-2, and iNOS immunostaining in hepatic tissue and restoration of serum NO.

### 3.5. Effect of α-LA on CsA-Induced Apoptosis in Rat Liver Tissues

**Figure 7:**
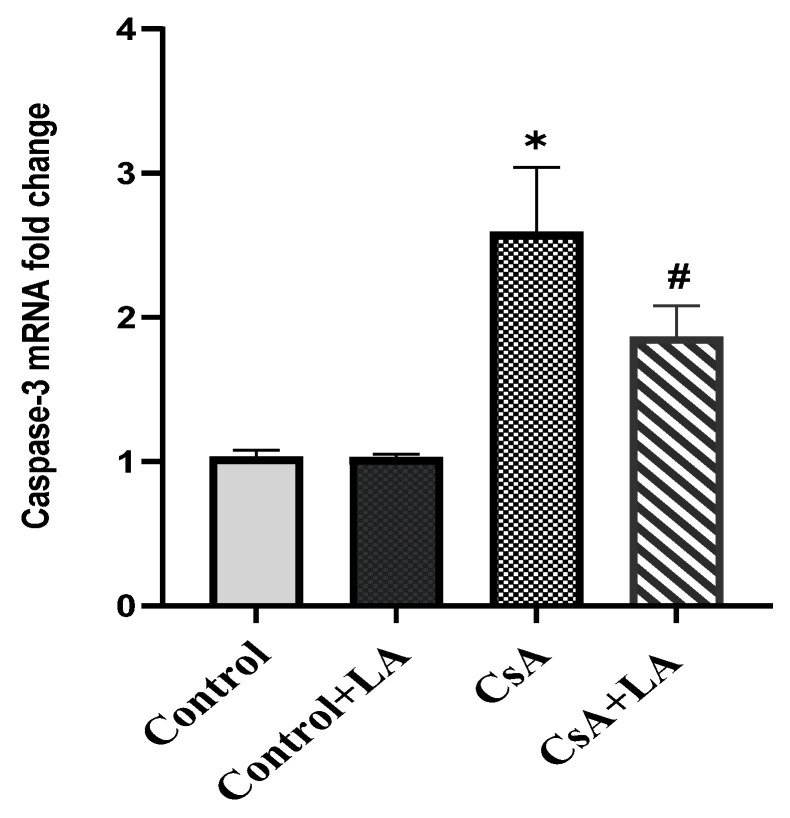
Effect of α-LA on caspase-3 mRNA fold change in hepatic tissues from various experimental groups. Data are expressed as mean ± SE. * the CsA-treated group versus the control group at *p* ˂ 0.05. ^#^ α-LA + the CsA-treated group versus the CsA-treated group at *p* ˂ 0.05.

**Figure 8:**
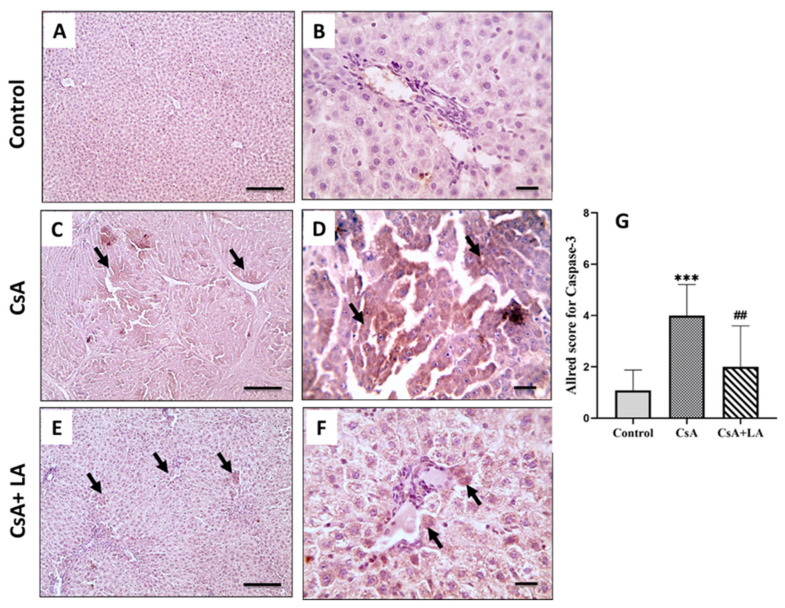
Microscopic pictures of immunostained hepatic sections against caspase-3. The control normal group (**A**,**B**) shows negative expression. Hepatic sections (**C**,**D**) from the CsA-treated group show prominent positive brown expression against caspase-3 (black arrows). Hepatic sections (**E**,**F**) from (CsA + LA) treated group show markedly decreased positive brown expression against caspase-3 in areas of perivascular necroinflammatory lesions (black arrows). IHC counterstained with Mayer’s hematoxylin, (**A**,**C**,**E**; Scale bar = 100 μm), and (**B**,**D**,**F**; Scale bar = 50 μm). (**G**) represents the Allred score for caspase-3 cytoplasmic expression. Data are expressed as mean ± SE (*n* = 8). *** *p* < 0.001 versus the control group, ^##^ *p* < 0.01 versus the CsA group. Allred index (0–1 = negative, 2–3 = mild, 4–6 = moderate, and 7–8 = strongly positive).

CsA administration induced apoptosis in rats’ hepatic tissue as indicated by a significant increase in caspase-3 mRNA expression and immunostaining in hepatic tissue compared to the control group. In contrast, administration of α-LA to CsA-treated rats significantly down-regulated caspase-3 mRNA expression and immunostaining in hepatic tissues, Figure 7 and Figure 8.

## 4. Discussion

The present study demonstrated protective efficacy of α-LA in CsA-induced liver injury. α-LA administration decreased oxidative stress and restored antioxidant element levels in serum and liver tissues, downregulated inflammatory cell recruitment and inflammatory markers, and suppressed apoptosis.

Clinical and experimental studies demonstrated that CsA treatment is associated with functional and morphological changes [1,29,30]. CsA is a prototypical cholestasis-producing agent. It affects hepatocytes, the mitochondrial function canalicular system, causing liver injury [1]. Hyperbilirubinemia, elevated serum transaminases, ALP, and GGT characterize functional alterations, while mononuclear cell infiltrations, congestion, and hepatocytes’ degenerative changes with nodular cirrhosis identify morphological changes [31]. Consistently, the results of the present study reported impaired liver function in parallel with microscopic lesions in the CsA-treated group.

Oxidative stress has been studied as the underlying pathogenic pathway in CsA-induced toxicity by several studies [32,33]. Indeed, CsA causes intramitochondrial Ca^++^ disturbance, disrupts mitochondrial oxidative phosphorylation, triggers ROS production, and induces oxidative damage to macromolecules [34]. These events were coupled with impaired intracellular antioxidant systems. This pro-oxidants/antioxidants imbalance disrupts normal cellular functioning and membrane integrity. In line, the present study demonstrated increased MDA and NO concomitant with compromised TAC in serum and SOD gene expression in the hepatic tissue of CSA-treated animals. This was associated with increased hepatic tissue MDA content along with reduced GSH levels, SOD, and CAT activity in CsA-treated animals.

Nrf2 is a transcription factor that regulates several genes encoding for detoxifying enzymes and antioxidant proteins such as HO-1, glutathione S-transferase, glutathione peroxidase modulation of NADPH oxidase, and the Nrf2/HO-1 pathway by vanillin in cisplatin-induced nephrotoxicity in rats [35,36]. Moreover, Nrf2 regulates the balance of apoptotic/antiapoptotic proteins, controlling cellular apoptosis [37]. HO-1 is one of the classical Nrf2 controlled genes and the Nrf2/HO-1 axis has been considered as a crucial antioxidant target. Further, it has been reported that activation of the Nrf2/HO-1 axis is accompanied by NF-κB inhibition in various models of liver toxicity [38,39]. Indeed, CsA-induced hepatorenal injury was accompanied by a decrease in Nrf2 tissue expression, along with its target antioxidant proteins, including HO-1 [40]. Similarly, our data showed reduced Nrf2 gene expression and HO-1 immunostaining in the liver tissue of CSA-treated animals.

Downregulation of Nrf-2 activates NF-κB with subsequent activation of pro-inflammatory mediator production, including COX-2 and inflammatory cytokines. On the other hand, activation of NF-κB disrupts Nrf-2 signaling [41]. Indeed, previous studies demonstrated increased hepatic NF-kB p65 and inflammatory cytokines levels in CsA-treated rats [42].

Constitutive production of NO plays a crucial role in hepatic perfusion. NO is produced by NOS, which possess three subtypes, inducible (iNOS), neuronal (nNOS), and endothelial (eNOS) [1]. Studies have shown that baseline production of NO by eNOS is hepatoprotective, however increased NO levels by iNOS in an inflammatory environment is damaging to hepatic tissues [43]. Of note, macrophages are considered the major source of iNOS. In this context, CsA treatment is accompanied by increased inflammatory cell infiltration along with upregulated iNOS in hepatic tissue. Interestingly, this was concomitant with a decrease in NO level, which can be explained by consumption of produced NO by oxidative stress [1]. In addition to iNOS, inflammatory cells also produce COX-2. Increased COX-2 expression triggers the production of vasoconstrictive agents, leading to a reduction in blood flow to tissues. Additionally, COX-2 and its products are generally considered potent proinflammatory mediators [44]. CsA treatment is accompanied by increased COX-2 levels in various tissues [45,46]. In agreement with these reports, the IHC findings of the present study demonstrated inflammatory cell infiltration as reflected by increased immunostaining of CD68, in addition to increased iNOS and COX-2 immunostaining in rat hepatic tissues following CsA administration.

Oxidative stress and inflammation are well-known inducers of apoptotic changes [47]. Previous reports indicated induction of cellular apoptosis by CsA treatment [48]. Caspase-3 is a main protease implicated in cellular apoptosis, where it is considered the last signal of cell death. CsA has been reported to enhance the expression and activation of caspase-3 leading to cell apoptosis [4,49,50]. In line, the CsA-treated group in the present study demonstrated increased hepatic mRNA levels and immunostaining of caspase-3.

Based on the above discussion, oxidative stress, inflammation, and apoptosis are considered key pathogenic pathways in CsA-induced toxicity to various body organs including the liver. Thereby, targeting these pathways could be an effective strategy to combat CsA- accompanied toxicity.

ALA and its reduced/dihydro metabolite have been reported as powerful inhibitors of lipid and protein oxidation and free-radical quenching agents. Interestingly, ALA is both fat and water soluble; allowing it to act as an antioxidant in both fatty and watery parts of cells [51]. In addition to its antioxidant efficacy, ALA has been widely studied for its anti-inflammatory and anti-apoptotic impacts in various disease models [13,52,53,54]. In this context, ALA demonstrated protective efficacy against CsA-induced renal [55], pancreatic [56], testicular [57], and neurological toxicity [58]. Similarly, ALA in the present study protected against CsA-induced liver injury through modulation of oxidative stress, inflammation, and apoptosis.

ALA protective efficacy has been attributed to its effect on energy metabolism and/or redox status. ALA enhances glucose uptake and protects mitochondrial function. In addition, ALA is a direct scavenger of reactive oxygen species, metal chelator and stabilizer, and inducer of cellular antioxidants [7,59]. Herein, we demonstrated that ALA could attenuate CsA-induced oxidative stress. However, the potential effect of ALA on energy metabolism needs to be investigated.

Previous studies have reported a high liver capacity for the uptake and accumulation of ALA. Administration of ALA has been associated with amelioration of energy-impaired and redox-unbalanced diseases [60]. On the other hand, Silvestri et al. reported that α-lipoic acid might have a double-edged behavior in terms of its oxidative state that may vary according to the biological compartment considered. Nevertheless, an ALA-associated increase in ROS level did not reach an extent able to promote oxidative DNA damage [61]. Furthermore, excessively high doses of ALA have been reported to induce cellular mitochondrial damage. This was attributed to the acceleration of aerobic respiration at high ALA doses, leading to the heating up of the mitochondria with subsequent breakdown of their membranes [62]. Therefore, ALA at high doses may be detrimental due to its deleterious effects on energy metabolism and redox status, whether this effect is a direct effect or due to the increased endogenous ALA synthesis needs further investigation.

## 5. Conclusions

In conclusion, the present study showed that the antioxidant, anti-inflammatory, and anti-apoptotic activities of ALA prevented CsA-induced liver injury. Thus, co-administration of ALA might represent an effective therapeutic strategy to ameliorate liver damage induced by CsA. However, further studies are needed to investigate the effect of ALA on mitochondrial function and energy consumption in CsA-induced liver injury. Additionally, whether this effect is a direct effect of administered ALA, or due to an enhanced endogenous production of ALA warrants further investigation.

## Figures and Tables

**Table 1:** Effect of α-LA administration on serum liver function tests in CsA-treated animals.

Parameter	Control	Control + α-LA	CsA	CsA + α-LA
ALT(IU/L)	41.7 ± 8.1	34.4 ± 9.5	54.1 ± 13.3 *	43.1 ± 14.2 ^#^
AST(IU/L)	54.1 ± 9.5	47.2 ± 11.1	77.2 ± 21.8 *	61.1 ± 2.4 ^#^
GGT(IU/L)	23.4 ± 7.2	22.3 ± 6.1	38.1 ± 1.7 *	29.6 ± 1.2 ^#^
ALP(IU/L)	63.5 ± 12.4	59.5 ± 11.5	75.3 ± 12.5 *	63.2 ± 12.2 ^#^

Data are expressed as mean ± SE, * CsA-treated group versus control group at *p* ˂ 0.05, ^#^ α-LA + CsA-treated group versus CsA-treated group at *p* ˂ 0.05.

**Table 2:** Effect of α-LA administration on serum oxidative stress in CsA-treated animals.

Parameter	Control	Control + α-LA	CsA	CsA + α-LA
TBARS (μmol MDA/L)	24.3 ± 4	22.7 ± 2.8	39.4 ± 9.3 *	28.7 ± 7.5 ^#^
TAC (mmol/L)	629.7 ± 157.8	693.8 ± 156.8	377 ± 98.9 *	600 ± 124.3 ^#^

Data are expressed as mean ± SE, * CsA-treated group versus control group at *p* ˂ 0.05, ^#^ α-LA+ CsA-treated group versus CsA-treated group at *p* ˂ 0.05.

**Table 3:** Effect of α-LA administration on serum oxidative stress in CsA-treated animals.

Parameter	Control	Control + α-LA	CsA	CsA + α-LA
TBARS (nmol/g.tissue)	12.6 ± 1.9	13.7 ± 2.5	35.9 ± 2.2 *	21.5 ± 1.2 ^#^
SOD (U/g.tissue)	139.8 ± 5.2	125.7 ± 11.2	69.08 ± 4.2 *	106.1 ± 4.7 ^#^
CAT (U/g.tissue)	5 ± 0.58	4.5 ± 0.37	1.7 ± 0.06 *	3.2 ± 0.09 ^#^
GSH (mmol/g.tissue)	2.1 ± 0.08	2.5 ± 0.03	0.88 ± 0.16 *	1.5 ± 0.15 ^#^

Data are expressed as mean ± SE, * CsA-treated group versus control group at *p* ˂ 0.05, ^#^ α-LA+ CsA-treated group versus CsA-treated group at *p* ˂ 0.05.

**Table 4:** Effect of α-LA administration on hepatic tissue mRNA expression of SOD and Nrf-2 genes in CsA-treated animals.

Parameter	Control	Control + α-LA	CsA	CsA + α-LA
SOD gene expression (2^−∆∆CT^)	0.99 ± 0.01	1.01 ± 0.02	0.53 ± 0.19 *	0.79 ± 0.23 ^#^
Nrf-2 gene expression (2^−∆∆CT^)	1.05 ± 0.04	1.03 ± 0.02	0.47 ± 0.11 *	0.88 ± 0.27 ^#^

Data are expressed as mean ± SE, * CsA-treated group versus control group at *p* ˂ 0.05, ^#^ α-LA + CsA-treated group versus CsA-treated group at *p* ˂ 0.05.

**Table 5:** Effect of α-LA administration on hepatic tissue mRNA expression of TNF-α and NF-κB genes and serum NO in CsA-treated animals.

Parameter	Control	Control + α-LA	CsA-Treated Group	CsA + α-LA
TNF-α gene expression (2^−∆∆CT^)	0.98 ± 0.03	0.01 ± 0.05	2.57 ± 0.64 *	1.61 ± 0.37 ^#^
NF-κB gene expression (2^−∆∆CT^)	1.03 ± 0.05	1.06 ± 0.04	2.38 ± 0.74 *	1.53 ± 0.41 ^#^
NO (μmol/L)	19.6 ± 4	20.2 ± 5	9.3 ± 3 *	17.9 ± 2 ^#^

Data are expressed as mean ± SE, * the CsA-treated group versus the control group at *p* ˂ 0.05, ^#^ α-LA + the CsA-treated group versus the CsA-treated group at *p* ˂ 0.05.

## Data Availability

The data presented in this study are available on request from the corresponding author. The data are not publicly available due to privacy.
